# Evaluating Shigella flexneri Pathogenesis in the Human Enteroid Model

**DOI:** 10.1128/IAI.00740-18

**Published:** 2019-03-25

**Authors:** Sridevi Ranganathan, Michele Doucet, Christen L. Grassel, BreOnna Delaine-Elias, Nicholas C. Zachos, Eileen M. Barry

**Affiliations:** a Center for Vaccine Development and Global Health, University of Maryland School of Medicine, Baltimore, Maryland, USA; b Department of Medicine, Division of Gastroenterology and Hepatology, Johns Hopkins University School of Medicine, Baltimore, Maryland, USA; University of Michigan-Ann Arbor

**Keywords:** human intestinal enteroids, M cells, *S. flexneri* pathogenesis, colonoid

## Abstract

The enteric pathogen *Shigella* is one of the leading causes of moderate-to-severe diarrhea and death in young children in developing countries. Transformed cell lines and animal models have been widely used to study *Shigella* pathogenesis. In addition to altered physiology, transformed cell lines are composed of a single cell type that does not sufficiently represent the complex multicellular environment of the human colon. Most available animal models do not accurately mimic human disease. The human intestinal enteroid model, derived from LGR5^+^ stem cell-containing intestinal crypts from healthy subjects, represents a technological leap in human gastrointestinal system modeling and provides a more physiologically relevant system that includes multiple cell types and features of the human intestine. We established the utility of this model for studying basic aspects of *Shigella* pathogenesis and host responses. In this study, we show that Shigella flexneri is capable of infecting and replicating intracellularly in human enteroids derived from different segments of the intestine. Apical invasion by S. flexneri is very limited but increases ∼10-fold when enteroids are differentiated to include M cells. Invasion via the basolateral surface was at least 2-log_10_ units more efficient than apical infection. Increased secretion of interleukin-8 and higher expression levels of the mucin glycoprotein Muc2 were observed in the enteroids following S. flexneri infection. The human enteroid model promises to bridge some of the gaps between traditional cell culture, animal models, and human infection.

## Abstract (precis)

The enteric pathogen *Shigella* is one of the leading causes of moderate-to-severe diarrhea and death in young children in developing countries. Transformed cell lines and animal models have been widely used to study *Shigella* pathogenesis.

## INTRODUCTION

*Shigella*, an enteroinvasive, Gram-negative bacterial pathogen, is the causative agent of shigellosis. *Shigella* causes significant morbidity and mortality, particularly in young children (<5 years old) and immunocompromised adults (1, 2). In 2010, the shigellosis mortality rate was estimated at ∼123,000 (3). Infection can be life threatening and is characterized by watery diarrhea followed by dysentery. A vast majority of the disease burden due to *Shigella* spp. can be attributed to Shigella flexneri in the developing world and to Shigella sonnei in more-industrialized regions. In the Global Enteric Multicenter Study (GEMS) that investigated *Shigella* isolates from pediatric cases across seven sites in south Asia and sub-Saharan Africa, S. flexneri accounted for ∼66% of cases (745 of 1,100) (1, 2). Antibiotics are administered as the standard-of-care treatment for shigellosis; however, the emergence of antibiotic-resistant strains is increasingly narrowing the available treatment options (4, 5).

S. flexneri has a low infectious dose: 10 to 100 bacteria are sufficient to cause infection (6). The bacterium spreads via the fecal-oral route upon ingestion of contaminated food or water and also via person-to-person contact. In the currently accepted paradigm, upon reaching the gastrointestinal tract, S. flexneri subverts the mucus layer by altering the expression and secretion of the mucin glycoproteins (7). The bacterium then triggers its own transcytosis via microfold (M) cells in the follicle-associated epithelium in the ileal and colonic mucosal surfaces (8, 9). S. flexneri is phagocytosed by resident macrophages residing at the basal pocket of M cells. However, the bacteria escape the phagocytic vacuole, induce pyroptosis of macrophages (10–12), and then invade epithelial cells via the basolateral surface (13). Multiplication (14, 15) and intercellular spread (16, 17) of S. flexneri within the epithelium trigger interleukin-8 (IL-8) secretion from epithelial cells and consequent neutrophil recruitment (18–20). S. flexneri and neutrophil-induced inflammation cause the destruction of the epithelium, leading to bloody mucoid diarrhea (21, 22). Eventually, the bacterial infection is cleared by neutrophils (23–25).

Researchers studying host-pathogen interactions have primarily relied on immortalized cell lines, such as HeLa, Caco-2, and T84, to understand diverse molecular pathogenesis processes. Immortalized cell lines have altered physiology and in some cases do not display appropriate polarization that is characteristic of intestinal epithelial cells. Also, transformed cell lines composed of a single cell type do not sufficiently represent the complex multicellular environment of the human intestine. One of the key paradigms of S. flexneri invasion is that the bacteria gain access to the basolateral surface by transcytosis via M cells. Studies have used elaborate coculture models using Caco-2 cells and murine Peyer’s patch lymphocytes or Raji B cells to recapitulate an M cell-like phenotype *in vitro* (26). However, these models require extensive manipulation and optimization to preserve the viability of host cells and achieve reproducibility. Primary cells overcome the drawback of altered physiology and better represent normal cell physiology. However, primary cells are short-lived and require continued sourcing. Researchers have also employed several animal models, such as nonhuman primate (27), young domestic pig (28), rabbit ligated ileal loop (9, 29), mouse pulmonary infection (30), guinea pig keratoconjunctivitis (31), and guinea pig rectocolitis (31) models, to evaluate host responses against *Shigella* infection. Most animal models replicate only a subset of characteristics observed in human infection. In a majority of animal models, no colonic colonization is observed (9, 28).

Human intestinal enteroids, derived from LGR5^+^ stem cell-containing colonic crypts from healthy subjects, represent a physiologically relevant model that includes some additional features of the human intestine compared to traditional cell culture models (32–34). Self-organizing and long-lived three-dimensional (3D) enteroids can be formed in a Matrigel matrix using defined growth factors to promote stem cell proliferation (35). The enteroids can also be propagated as monolayers on permeable support filters to facilitate access to both the apical and basolateral surfaces of the epithelium. Enteroid monolayers can be differentiated to a multicellular environment comprised of goblet cells, enterocytes, Paneth cells, and enteroendocrine cells. Enteroids derived from different sections of the intestine retain the unique functional properties (segmental specificity) of the region and exhibit apical and basolateral polarity with appropriate localization of surface markers and transporter proteins (34, 36–38). These features make enteroids a very attractive model to study host-pathogen interactions. Enteroids can be instrumental in understanding the very early steps in pathogenesis, such as interaction of bacterial pathogens with mucus, antimicrobial peptides, and different regions of the intestine (33, 39). As enteroids are derived from human tissues, they are expected to more closely mimic toxicity, efficacy, and safety concerns of drugs and vaccine candidates in human trials than animal models, thereby making them a very useful preclinical model that will bridge some gaps between cell culture/animal studies and human trials. However, it is important to acknowledge the limitations of this model system in its current state. In the enteroid model used in this study, components such as the host immune system, the gut microbiome, and peristalsis, etc., are not captured.

In this study, we establish the basic parameters of S. flexneri infection in the human enteroid model. We show that S. flexneri exploits M cells as a port of entry to penetrate the epithelial barrier and infects epithelial cells more efficiently via the basolateral surface. S. flexneri is able to replicate intracellularly in enteroids derived from different segments of the human intestine, and infection resulted in increased production of the mucin glycoprotein Muc2 and increased secretion of IL-8.

Further underscoring the utility and reproducibility of human intestinal enteroids for the study of *Shigella* pathogenesis is the work of Koestler et al. (40) that was performed in parallel with our study. Although there are some slight differences in protocols, both studies show invasion and replication of enteroids by S. flexneri, and each study provides new information on the host response to S. flexneri invasion. These studies serve as technical reports assessing the utility of human enteroids as a model to study bacterial pathogenesis.

## RESULTS

### Apical versus basolateral invasion of enteroids.

**FIG 1:**
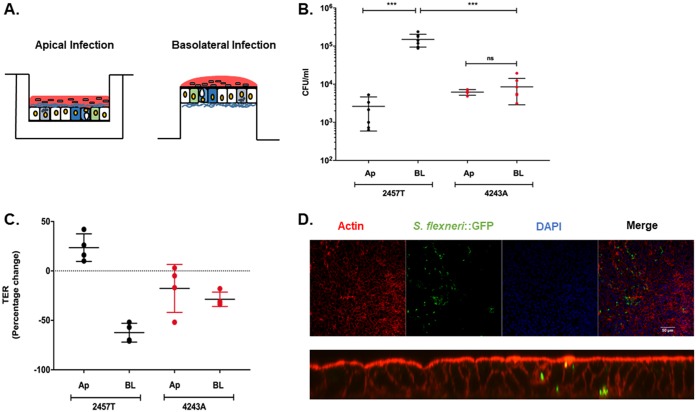
Apical versus basolateral invasion of enteroid monolayers. (A) Schematic representation of apical and basolateral routes of infection of enteroid monolayers grown on permeable transwell inserts. (B) Differentiated human enteroid monolayers (derived from colon) were infected either apically (Ap) or basolaterally (BL) with ∼4 × 10^7^ CFU of S. flexneri WT strain 2457T or plasmidless strain 4243A for 2 h. After gentamicin treatment to remove extracellular bacteria, monolayers were lysed to enumerate intracellular bacteria. The data presented are pooled from three independent experiments. Each dot represents data collected from an individual monolayer (*n* = 7 for all groups except for apical infection with the 4243A strain, where *n* = 4). Error bars indicate standard deviations from the means. The asterisks above the bars indicate statistically significant differences calculated using one-way ANOVA. ***, *P* < 0.0001 with Bonferroni’s multiple-comparison test. (C) Transepithelial electrical resistance (TEER) of enteroid monolayers was measured prior to and after infection. The percent change in TEER is plotted (see Materials and Methods for the formula). The data presented are pooled from two independent experiments. Error bars indicate standard deviations from the means. (D) Enteroid monolayers were infected basolaterally with ∼4 × 10^7^ CFU 2457T::GFP. Intracellular bacteria were visualized using confocal microscopy. (Top) *xy* projection; (bottom) *xz* projection. Actin was stained with an Alexa Fluor 555-conjugated phalloidin probe (red), S. flexneri::GFP was visualized by constitutive expression of green fluorescent protein (green), and nuclei were stained with DAPI (4′,6-diamidino-2-phenylindole) (blue).

We tested the abilities of wild-type (WT) S. flexneri serotype 2a strain 2457T and the plasmid-cured noninvasive derivative strain 4243A to invade enteroid monolayers (derived from colon) from the apical and basolateral surfaces. Differentiated enteroid monolayers were infected either apically or basolaterally with the WT or 4243A (see the schematic in Fig. 1A). Bacteria were incubated with enteroids for 2 h, followed by gentamicin treatment for 30 min to remove extracellular bacteria. At 2.5 h postinfection, comparable, low-level, apical invasion was observed for both the WT and 4243A (Fig. 1B). In contrast, higher-level invasion by WT *Shigella* occurred via the basolateral surface; at least 2-log_10_-higher numbers of WT intracellular bacteria were enumerated 2.5 h after basolateral invasion than after apical invasion (Fig. 1B). Increased basolateral invasion by WT *Shigella* was observed consistently in multiple experiments, with relative differences of 163-, 31-, and 60-fold increases over apical infection. A very modest increase in invasion was observed with 4243A, as almost equal numbers of intracellular bacteria were recovered from both invasion routes. The plasmid-cured 4243A strain showed at least a 10-fold-lower basolateral invasion efficiency than the WT (Fig. 1B). The results were confirmed in similar experiments performed using enteroids derived from a second healthy individual (data not shown).

The transepithelial electrical resistance (TEER) of the enteroid monolayers was measured prior to and after invasion (Fig. 1C). Apical infection with the WT strain resulted in a slight increase in TEER at 2.5 h postinfection (∼23%). In contrast, basolateral infection with the WT strain effected a steep drop in TEER (∼62%). Following infection with 4243A, a slight decrease in TEER was observed with both apical and basolateral infections. It is interesting to note that basolateral infection with 4243A did not result in a steep decrease in TEER as observed with the WT.

The S. flexneri 2457T::GFP strain was used to visualize intracellular bacteria in enteroids. A standard gentamicin protection assay quantifying intracellular bacteria by plate counts was performed to confirm that 2457T::GFP invaded enteroid monolayers with an efficiency equal to that of the unlabeled 2457T strain (data not shown). Following basolateral infection of enteroids, intracellular 2457T::GFP bacteria were confirmed using confocal microscopy (Fig. 1D).

### Uptake of S. flexneri by M cells.

**FIG 2:**
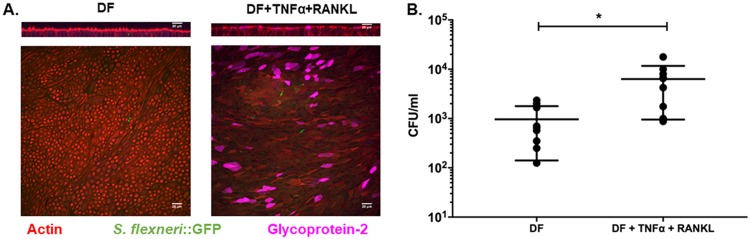
Uptake of S. flexneri via M cells. (A) Enteroid monolayers derived from the ileum were differentiated (DF) with or without TNF-α and RANKL in the basolateral medium to induce M cell differentiation. Differentiated monolayers were infected apically with ∼4 × 10^7^ CFU 2457T::GFP for 2 h, followed by gentamicin treatment to remove extracellular bacteria. Immunostaining for the human M cell marker glycoprotein 2 (GP2) confirmed induction of M cells in the enteroid monolayers. (Top) Representative *xz* plane; (bottom) maximum-intensity projection image. (B) Differentiated enteroid monolayers without or with M cells were infected apically with ∼4 × 10^7^ CFU WT S. flexneri for 2 h, extracellular bacteria were removed by gentamicin treatment, and intracellular CFU were enumerated by lysis of the monolayers and serial dilution plating of monolayer lysates. The data presented are pooled from three independent experiments. Each dot represents data collected from an individual enteroid monolayer (*n* = 6 in each group). Error bars denote standard deviations from the means. The asterisks above the bars indicate statistically significant differences determined using two-tailed, unpaired Student’s *t* test. *, *P* < 0.05.

The current paradigm holds that S. flexneri is taken up by M cells in the intestine. Previous work has shown that M cells can be generated in murine and human enteroids by stimulation with RANKL (receptor activator of NF-κB ligand) (41, 42) and that the RANKL-dependent increases in levels of M cell-related transcripts in mouse enteroids can be further enhanced by exposure to tumor necrosis factor alpha (TNF-α) (43). Therefore, we used RANKL and TNF-α to induce M cell differentiation in human ileal enteroids. The presence of M cells in the enteroid monolayers was confirmed using immunostaining for glycoprotein 2 (GP2) (Fig. 2A). Enteroid monolayers without or with M cells were infected apically with S. flexneri 2457T. Approximately 10-fold more intracellular bacteria were recovered from enteroid monolayers with M cells than from monolayers without M cells (Fig. 2B). Increased apical invasion in the presence of M cells was observed consistently in multiple independent experiments, with relative differences of 9-, 5-, and 6-fold increases over apical invasion of enteroids without M cells. To ensure that the increased number of intracellular CFU observed in the monolayers containing M cells was not due to compromised barrier integrity of the monolayer, the monolayers were immunostained for the tight junction protein ZO-1. Well-defined ZO-1 staining was observed in monolayers with M cells (see Fig. S1 in the supplemental material), confirming that the barrier integrity of the enteroid monolayers was uncompromised.

### Segmental specificity and intracellular replication.

**FIG 3:**
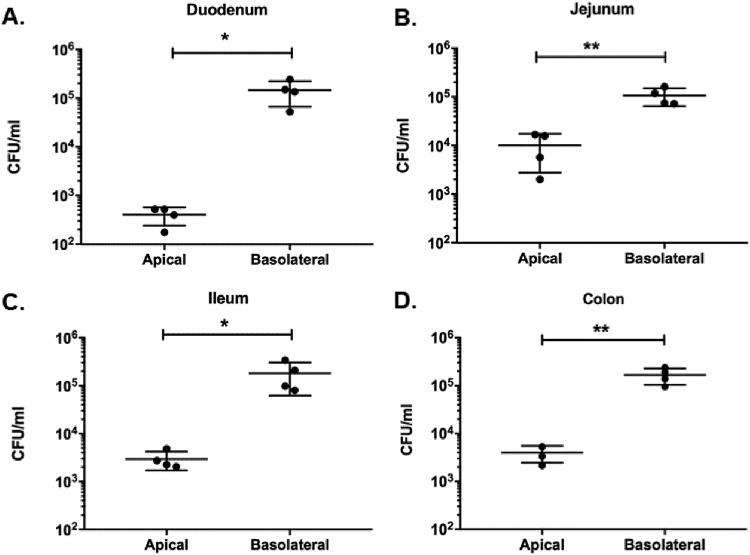
Segmental specificity of WT S. flexneri infection. Enteroid monolayers derived from four different segments of the human intestine were infected either apically or basolaterally with ∼4 × 10^7^ CFU WT S. flexneri 2457T for 2 h. Following gentamicin treatment to remove extracellular bacteria, the enteroid monolayers were lysed, and intracellular bacteria were enumerated. Intracellular numbers of *Shigella* bacteria are shown for enteroids derived from duodenum (A), jejunum (B), ileum (C), and colon (D). The data presented are pooled from 2 independent experiments. Each dot represents data collected from an individual enteroid monolayer (*n* = 4 in each group). Error bars indicate standard deviations from the means. The asterisks above the bars indicate statistically significant differences determined using two-tailed, unpaired Student’s *t* test. *, *P* < 0.05; **, *P* < 0.005.

Early studies using the rhesus macaque model of shigellosis showed electrolyte secretion abnormalities in the jejunum as well as the colon. However, colonization was observed primarily in the colon (44). We investigated the ability of WT S. flexneri to invade enteroid monolayers derived from different segments of the human intestine, namely, the duodenum, jejunum, ileum, and colon, both apically and basolaterally. WT S. flexneri was able to infect enteroids derived from all segments of the intestine via the apical and basolateral surfaces; invasion via the basolateral surface was more efficient in all segments (Fig. 3A).

**FIG 4:**
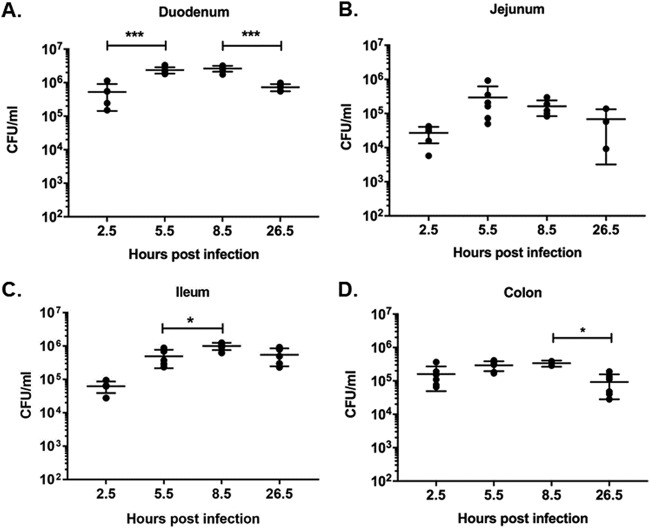
Intracellular replication of WT S. flexneri in enteroid monolayers. Enteroid monolayers derived from four different segments of the human intestine were infected basolaterally with ∼4 × 10^7^ CFU WT S. flexneri 2457T for 2 h. The monolayers were then incubated in medium containing gentamicin to remove extracellular bacteria. Intracellular CFU were enumerated by lysing and plating the monolayers at 2.5, 5.5, 8.5, and 26.5 h postinfection. Intracellular replication is shown for enteroid monolayers derived from duodenum (A), jejunum (B), ileum (C), and colon (D). The data presented are pooled from two independent experiments for enteroids derived from duodenum and ileum. For enteroids derived from jejunum and colon, data shown are from three independent experiments. Each dot represents data collected from an individual enteroid monolayer (*n* is variable between different time points). Error bars denote standard deviations from the means. The asterisks above the bars indicate statistically significant differences determined using one-way ANOVA with Bonferroni’s multiple-comparison test. *, *P* < 0.05; ***, *P* < 0.0001.

The ability of S. flexneri 2457T to replicate intracellularly in enteroids derived from the four different segments of the intestine was measured. The enteroids were infected basolaterally for 2 h, followed by gentamicin treatment to remove extracellular bacteria. Gentamicin was maintained in the medium during subsequent incubation periods to prevent reinfection. Intracellular bacteria were quantitated at four different time points postinfection (2.5, 5.5, 8.5, and 26.5 h). WT S. flexneri was able to replicate intracellularly in enteroids derived from all four segments (Fig. 4). Different bacterial doubling rates were observed in the enteroids derived from the four segments of the intestine: duodenum (range of doublings observed between biologically independent experiments of 1.8 to 3.4), jejunum (0.4 to 2.1), ileum (2.4 to 4.1), and colon (0.7 to 1.2 in one line and 1.6 to 2.9 in a second line). Surprisingly, a very low doubling rate was observed in the enteroid monolayers derived from colons of two donors.

### Interleukin-8 secretion.

**FIG 5:**
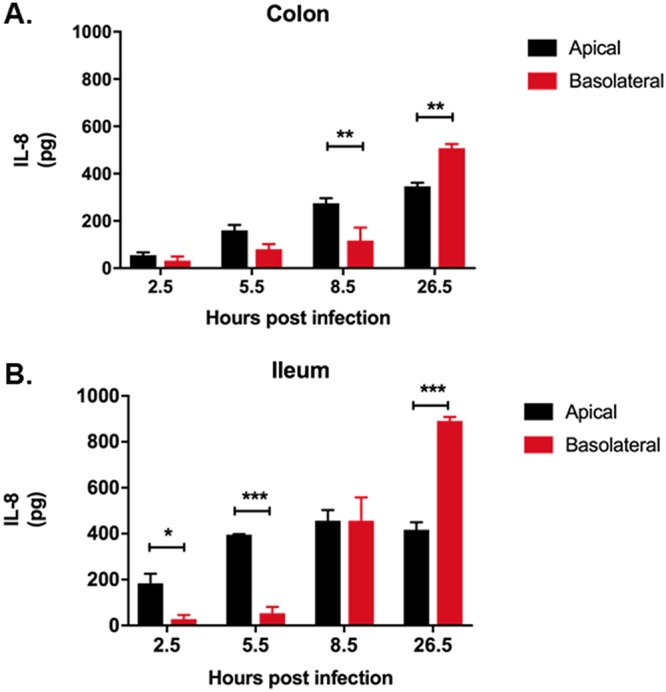
Effect of S. flexneri infection and intracellular replication on interleukin-8 expression. Enteroid monolayers derived from colon and ileum were infected basolaterally with ∼4 × 10^7^ CFU WT S. flexneri 2457T for 2 h. Following infection, the monolayers were incubated in gentamicin-containing medium for 30 min to remove extracellular bacteria. Apical and basolateral supernatants were collected at 2.5, 5.5, 8.5, and 26.5 h postinfection. The mean levels of IL-8 secreted in apical and basolateral supernatants in enteroids derived from colon (A) and ileum (B) were quantified by ELISAs. The data presented are pooled from two independent experiments with at least 2 enteroid monolayers in each experiment. Error bars denote standard deviations from the means. The asterisks above the bars indicate statistically significant differences determined using two-way ANOVA with Bonferroni’s multiple-comparison test. *, *P* < 0.05; **, *P* < 0.005; ***, *P* = 0.0005.

Induction of IL-8 secretion is a hallmark of *Shigella* infection (19, 45). We measured the amount of IL-8 secreted by infected enteroid monolayers into the apical and basolateral supernatants. Supernatants were collected from enteroid monolayers infected basolaterally with WT S. flexneri for 2 h and then incubated with gentamicin-containing medium for 30 min (considered 2.5 h postinfection) or 5.5 h, 8.5 h, or 26.5 h to allow intracellular bacterial replication. Increasing IL-8 secretion in apical and basolateral supernatants from both ileum- and colon-derived enteroids infected with WT S. flexneri was quantified in a time-dependent manner (Fig. 5A and B). Supernatants from the apical compartment revealed higher levels of IL-8 than in basolateral supernatants at 2.5 and 5.5 h postinfection in enteroids derived from the ileum (Fig. 5). At 8.5 h postinfection, the level of apical IL-8 was significantly higher than the level of basolateral IL-8 in enteroids derived from the colon. At 26.5 h postinfection, the level of basolateral IL-8 was higher than the level of apical IL-8 in infected enteroids derived from either segment.

### Effect of S. flexneri invasion on mucus expression.

**FIG 6:**
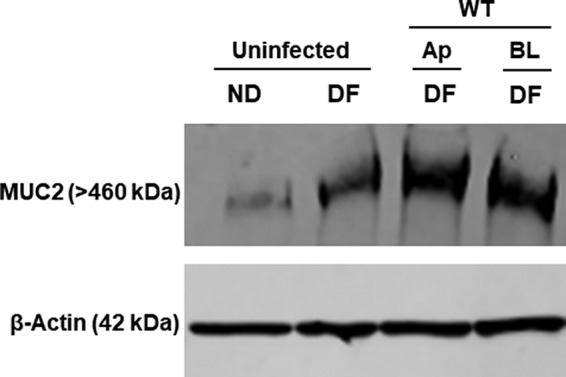
Effect of S. flexneri infection on mucus expression. Enteroid monolayers derived from the colon were infected either apically or basolaterally with 4 × 10^7^ CFU of WT S. flexneri for 2 h. Following invasion, the enteroid monolayers were incubated in medium containing gentamicin for an additional 4 h to allow intracellular replication of bacteria and to remove extracellular bacteria. Undifferentiated (ND) and differentiated monolayers (both uninfected) were used as controls. Whole-cell lysates of uninfected or infected enteroids were probed for Muc2 by Western blotting. A Western blot with β-actin is shown to confirm equal loading of samples (bottom panel).

Mucus forms a protective barrier on the gut mucosal surface and is composed of nonoligomerizing and oligomerizing gel-forming mucins (46). One advantage of the human enteroid model is the expression of physiologically relevant mucus. A previous study demonstrated that *Shigella* infection resulted in transcriptional upregulation of the oligomerizing gel-forming mucin Muc2 in the transformed HT29-MTX cell line (7). We examined the effect of apical and basolateral S. flexneri infection on mucus expression in enteroids. As shown previously (33), differentiated enteroids expressed a larger amount of Muc2 than did undifferentiated enteroids (Fig. 6). WT S. flexneri infection from both the apical and basolateral routes resulted in increased expression of Muc2.

## DISCUSSION

S. flexneri manipulates host immune defenses at various stages of its pathogenic process to successfully establish an infection. Cell culture and animal infection models have been instrumental in identifying mechanisms of S. flexneri pathogenesis. However, there are gaps in our understanding, especially of the early infection steps, such as mucus barrier penetration, evasion of antimicrobial peptides, and uptake by M cells, vastly due to the lack of appropriate models to study these processes. The human enteroid model promises to serve as a novel model to help fill some of these knowledge gaps. In this study, we lay the groundwork for using this exciting new model to study S. flexneri infection.

S. flexneri has a limited ability to infect epithelial cells from the apical surface (13) (Fig. 1 and 3). Many enteric pathogens, such as S. flexneri, Vibrio cholerae (47), *Salmonella* spp. (48, 49), Listeria monocytogenes (50), and viruses such as poliovirus (51) and reovirus (52), target M cells as a route of entry to penetrate the epithelial barrier from the gut lumen. M cells serve as ports of antigen sampling in the lumen, as they reside above the lymphoid follicle domes or Peyer’s patches (reviewed in references 53 and 54). The relatively thin glycocalyx on the surface of M cells compared to neighboring enterocytes (55) and their high endocytic activity and decreased levels of lysosomal and hydrolytic enzymes (56) make them vulnerable to exploitation by enteric pathogens.

Animal infection models provided the first evidence of S. flexneri’s association with M cells. Rhesus macaques infected with a S. flexneri *icsA* mutant, which lacks the ability to spread from cell to cell, developed attenuated disease with localized infection in the gut-associated lymphoid follicles (57). The rabbit ligated ileal loop model showed similar results, with entry of S. flexneri occurring preferentially via M cells in Peyer’s patches (9, 21). It is now known that the number and distribution of M cells as well as the carbohydrates expressed on the M cell surface vary among different host species and also between anatomical regions within the same host (53, 58), thereby making it important to study M cells derived from the homologous host. It was shown that RANKL stimulation of human enteroid monolayers resulted in a robust increase in the number of GP2-positive M cells (41) and that TNF-α increased the levels of M cell-related transcripts in mouse enteroids (41). In our study, based on this information, we used TNF-α and RANKL to induce M cell differentiation in ileal enteroid monolayers (41, 43). Approximately 10-fold more intracellular S. flexneri bacteria were enumerated after apical infection of enteroid monolayers containing M cells. Using confocal microscopy, we observed GP-2-positive M cells in the enteroid monolayers. However, intracellular S. flexneri bacteria were visualized in non-M cells. We reason that this is a result of the long infection time (2 h of infection followed by 30 min of gentamicin treatment) and high inoculum concentration (∼4 × 10^7^ CFU) used in our experiments. A previous study that established M cell induction in enteroid monolayers also observed intracellular Salmonella enterica serovar Typhimurium in non-M cells and M cells when enteroid monolayers were infected with *S*. Typhimurium at an inoculum concentration of 10^7^ CFU. One of the key advantages of the human enteroid model is the ability to induce the differentiation of diverse cell types, such as M cells, goblet cells, Paneth cells, and enteroendocrine cells, in an *in vitro* setting. Treatments with TNF-α and RANKL are also likely to have other signaling effects on the enteroids apart from the differentiation of M cells. With further characterization of the functional properties of these M cells and their ability to transcytose cargo, this model could be very useful in studying early steps in pathogenesis that have been elusive to date.

The three segments of the small intestine, namely, the duodenum, jejunum, and ileum, and the large intestine or colon express distinct sets of proteins that enable them to carry out their specialized functions (59–63). As adult stem cells in each intestinal segment are intrinsically programmed to have segment-specific functions, it is expected that enteroids derived from different segments of the murine or human intestine will retain the properties of the parent segment in long-term culture (36). This provides a model system where segment-specific pathology can be tested. Enteroaggregative Escherichia coli infection of enteroids showed distinct segment-specific aggregation phenotypes of the pathogen (64). Rotavirus successfully infected enteroids derived from all segments of the small intestine, which correlates with clinical disease in humans (65). In contrast, some researchers have reported an apparent lack of segmental specificity. For example, Clostridium difficile, which causes colitis in humans, was able to infect organoids that more closely represent the small intestinal environment (66). In this study, we show that WT S. flexneri, which predominantly causes colonic pathology, was able to infect enteroids derived from all segments of the intestine with comparable efficiencies when delivered to the basolateral surface (Fig. 3). This suggests to us that, after gaining access to the basolateral epithelial cell surface, S. flexneri can infect all intestinal segments and that the specificity of human infection in the colon may be a result of apical interactions. This also highlights that components of the gut intestine, such as the microbiome, intact mucus layer, antimicrobial peptides, mechanical forces from lumenal flow and peristalsis, and secretions such as bile, that are not included in the enteroid model at present may have a considerable impact on the segment-specific pathology observed in human disease. We recently reported the functional incorporation of macrophages in the enteroid system to better model the *in vivo* gut environment (32). Microfluidic devices (gut on a chip) that can include components such as the microbiome and endothelial vasculature and mimic physical forces such as peristalsis would be key in addressing some of the limitations of the current enteroid model (67, 68). Based on the ongoing efforts in many laboratories to optimize and advance this model, we anticipate its widespread use for the study of human-specific pathogens and host responses.

S. flexneri was able to replicate in enteroids derived from all four segments of the intestine albeit with different replication rates (Fig. 4). Segment-specific as well as donor-specific properties are likely to contribute to the differences in intracellular replication rates. We observed a lower-than-expected replication rate in enteroids derived from the colon, the site associated with acute pathology during shigellosis (Fig. 4D). This is not likely due to donor-specific characteristics, as similar results were observed in enteroids derived from another donor colon sample (data not shown). Based on our results, we speculate that colonic enteroids may be hypersusceptible to S. flexneri infection, in which fewer intracellular bacteria are capable of inducing apoptotic epithelial cell death. This might also in part explain the destructive colonic pathology associated with the disease. Another possible explanation for the low intracellular replication rate could be a partial inhibitory effect of gentamicin on intracellular bacteria with extended incubation. We used 50 μg/ml of gentamicin in all experiments, based on our observation that equal numbers of intracellular bacteria were recovered after treatment of monolayers with either 10 μg/ml or 50 μg/ml gentamicin at early infection time points (data not shown). However, the effects of gentamicin on the human enteroid model over extended periods of time have not been studied. Therefore, it cannot be ruled out that extended incubation times such as those used in intracellular replication studies might have had an inhibitory effect on intracellular bacteria, resulting in a lower replication rate.

Very little is known about how S. flexneri subverts the mucus barrier to gain access to the apical surface of the epithelium. Virulent S. flexneri altered the transcription of genes encoding various mucin glycoproteins in infected HT29-MTX cells (7). S. flexneri infection altered the extracellular secretion of another gel-forming mucin glycoprotein, Muc5Ac to allow for more-efficient invasion of the bacteria in HT29-MTX cells (7). In this study, we show that infection with WT S. flexneri results in increased expression of the mucin glycoprotein Muc2 at 4 h postinfection. This roughly correlates with the transcriptional increase in the level of *muc2* observed in HT29-MTX cells infected with S. flexneri M90T at 3 h postinfection. In our studies, apical or basolateral infection with WT S. flexneri resulted in increased Muc2 expression by enteroid monolayers, suggesting that either only low-level invasion is required or secreted bacterial effectors may play a role in altered mucin expression, as apical invasion is very inefficient. Recent studies showed a drastic decrease in Muc2 production by enteroids upon infection with enterohemorrhagic E. coli (EHEC) (33). The induction of mucus production may be a protective response by the host, as S. flexneri bacteria are nonflagellated, and the viscous mucus might further compromise bacterial mobility. Alternatively, bacterial effectors might be able to skew mucus production to glycoproteins that can be easily degraded or more favorably metabolized as carbon sources, thereby hijacking a host innate immune defense mechanism.

S. flexneri interaction with the basolateral surface of epithelial cells induces the production of large amounts of IL-8 (18, 19, 45). IL-8 acts as a chemokine recruiter of polymorphonuclear leukocytes (PMNs) to the site of infection (18). Transmigration of PMNs across the epithelial lining in the basolateral-to-apical direction contributes to the destruction of the epithelial barrier, aiding bacterial translocation (19). T84 cells plated and differentiated on permeable filters and infected with S. flexneri produced increased IL-8 levels in the basolateral supernatant (69). In our studies, using human enteroids derived from colon and ileum, we observe an increase in the IL-8 level in the basolateral compartment over time (Fig. 5). We also observe an increase in the IL-8 level in the apical supernatant. Considering that enteroids more closely mimic normal human physiology, this knowledge about apical secretion of IL-8 during an early phase of S. flexneri infection may provide additional insight into the pathogenic process and be useful in modeling host responses and disease.

Along with accurately recapitulating existing knowledge about S. flexneri pathogenesis, the human enteroid model also serves as a platform to further our understanding of host-pathogen interactions. We anticipate that future studies will assess the utility of human intestinal enteroids as innovative preclinical models for the evaluation of novel therapeutics and vaccine candidates and accelerate the advancement of interventional strategies against this important pathogen.

## MATERIALS AND METHODS

### Tissue collection and enteroid generation.

Human enteroid cultures were established from biopsy specimens obtained after endoscopic or surgical procedures by utilizing methods developed by the laboratory of Hans Clevers (35). Deidentified biopsy tissue was obtained from healthy subjects who provided informed consent at Johns Hopkins University, and all methods were carried out in accordance with approved guidelines and regulations. All experimental protocols were approved by the Johns Hopkins University Institutional Review Board (IRB) (approval number NA_00038329).

### Enteroid medium composition.

All enteroid media were prepared as reported previously (32). Advanced Dulbecco’s modified Eagle’s medium (DMEM)–F-12 medium supplemented with 1× GlutaMAX (Gibco), 10 mM HEPES (Quality Biologicals), and 100 Units/ml penicillin-streptomycin (Gibco) was used as the basal medium.

Complete medium with growth factor (CMGF^+^) is comprised of 22.3% (vol/vol) basal medium, 50% (vol/vol) Wnt3a-conditioned medium, 15% (vol/vol) R-spondin-1-conditioned medium, 10% (vol/vol) noggin-conditioned medium, 1× B27 supplement (Gibco), 1 mM *N*-acetylcysteine (Sigma), 1× Primocin (InvivoGen), 50 ng/ml human epidermal growth factor (R&D Systems), 10 nM [Leu-15]-gastrin (AnaSpec), 500 nM A83-01 (Tocris), and 10 μM SB202190 (Tocris).

Differentiation medium is comprised of 86.2% (vol/vol) basal medium, 10% (vol/vol) noggin-conditioned medium, 1 mM *N*-acetylcysteine (Sigma), 50 ng/ml human epidermal growth factor (R&D Systems), 10 nM [Leu-15]-gastrin (AnaSpec), and 500 nM A83-01 (Tocris).

### Enteroid monolayers.

Enteroids isolated from intestinal crypt cells were cultured as 3D cysts embedded in Matrigel (Corning) and passaged approximately every 7 to 10 days. 3D enteroids were harvested by incubation in an organoid harvesting solution (Cultrex) and gentle scraping, followed by vigorous shaking at 4°C for 30 min. The enteroids were triturated 25 to 30 times, and the contents of multiple wells were pooled in a 15-ml conical tube. The fragmented enteroids were washed using an equal volume of basal medium. The enteroids were collected by centrifugation at 1,200 rpm for 10 min at 4°C.

For passaging, the enteroid pellet was resuspended in Matrigel and seeded such that each split contained at least 50 enteroids. The plate was incubated at 37°C for 5 to 10 min to allow the Matrigel to polymerize. A total of 0.5 ml of CMGF^+^ containing 10 μM each Y-27632 (Tocris) and CHIR99021 (Tocris) was added to each well. The medium was replaced with CMGF^+^ without Y-27632 and CHIR99021 after 48 h. Fresh CMGF^+^ was added to the wells every other day.

To form enteroid monolayers, the triturated enteroids were resuspended in CMGF^+^ containing Y-27632 and CHIR99021 (32, 33). Transparent polyester membrane 24-well cell culture inserts with a 3.0-μm or 0.4-μm pore size (transwell filters; Corning) were precoated using 100 μl of a 34-μg/ml human collagen IV solution (Sigma) by incubation at 37°C for a minimum of 2 h or at 4°C overnight. For basolateral bacterial invasion assays, a 3.0-μm-pore-size filter was used to allow unobstructed passage of bacteria through the filter to the basolateral surface of the colonoid monolayer. For apical invasion assays, 3.0-μm- or 0.4-μm-pore-size transwells were used. Collagen-coated transwells were washed with basal medium before use. One hundred microliters of resuspended enteroid fragments was added to the transwells. Six hundred microliters of CMGF^+^ with Y-27632 and CHIR99021 was added to the receiver well. Cultures were incubated at 37°C with 5% CO_2_.

Typically, confluence in these colonoid cultures was achieved in 10 to 14 days. Monolayer confluence was assessed by the increase in transepithelial electrical resistance (TEER) measured using an epithelial volt/ohm meter (EVOM^2^; World Precision Instruments). Confluent monolayers were differentiated by incubation with Wnt3A-free and R-spondin-1-free medium (differentiation medium) for 5 days. Bacterial invasion assays were typically performed at 5 days postdifferentiation.

To differentiate enteroid monolayers to contain M cells, basolateral differentiation medium was supplemented with 50 ng/ml TNF-α and 100 ng/ml RANKL. The medium was changed every other day. Bacterial invasion assays were typically performed at 5 days postdifferentiation.

The percent change in TEER was calculated using the following formula, where 0.33 cm^2^ is the surface area of the transwell used: [(TEER_postinvasion_ × 0.33) − (TEER_preinvasion_ × 0.33)]/(TEER_preinvasion_ × 0.33) × 100%.

### Bacterial strains.

The following S. flexneri strains were used in this study: wild-type S. flexneri serotype 2a strain 2457T, a plasmid-cured S. flexneri serotype 2a strain derived from the 2457T strain (referred to as 4243A), and S. flexneri serotype 2a strain 2457T with green fluorescent protein (GFP) integrated into the chromosome (2457T::GFP) (see details below). All the strains were grown on Trypticase soy agar (TSA) plates (BD Difco) containing 0.01% Congo red dye. All bacterial strains were grown at 37°C unless specified otherwise.

### Construction of 2457T::GFP.

The OmpC_GFP fragment (encoding GFP driven by the *ompC* promoter) was generated by PCR from pGEN206 (70) using primers Wu255_OmpC_F2 (5′-TAACCTAGGGAATTCTGTGGTAGCACAGAATAATG-3′) and Wu255_OmpC_R2 (5′-TAATTAATTAACCGAGAAAAAAAAGCCCGCTCATTAG-3′). Vector plasmid pGRG25-mlpp (71) and the above-described PCR product were digested with PacI and AvrII (New England Biolabs). The restriction-digested PCR product OmpC_GFP was cloned into pGRG25-mlpp to generate the pGRG25-OmpC_GFP construct. The plasmid DNA of pGRG25-OmpC_GFP was electroporated into S. flexneri serotype 2a strain 2457T. 2457T(pGRG25-OmpC_GFP) was grown in tryptic soy broth at 30°C supplemented with 0.21% arabinose to induce Tn*7* transposase expression. The strain was then subcultured in tryptic soy broth at 37°C without ampicillin to cure the plasmid. The broth culture was serially diluted and plated onto TSA plates to generate individual colonies. Colonies were screened by PCR using primers Wu215_F (5′-ACCGAACAACGAACTGTTGGAA-3′) and Wu215_R (5′-TGCGTAGCGTTACAGTACCTGAT-3′) to confirm the integration of OmpC_GFP into the 2457T chromosome.

### Gentamicin protection assay.

Invasion assays were carried out using differentiated enteroid monolayers. Bacterial inocula were prepared in Dulbecco’s phosphate-buffered saline (DPBS) by resuspending a loopful of bacteria from TSA plates. The bacterial suspension was diluted to the desired bacterial concentration (typically 5 × 10^8^ CFU/ml) in DMEM without serum. For apical invasion assays, 75 μl of the inoculum (∼4 × 10^7^ CFU) was added onto the enteroid monolayers in the cell culture inserts. For basolateral invasion assays, the cell culture inserts were inverted onto a sterile petri dish, and 75 μl of the bacterial inoculum was carefully added as a drop on the basolateral side. The infected monolayers were incubated at 37°C with 5% CO_2_ for 2 h to allow bacterial invasion of enteroid monolayers. Following 2 h of invasion, the culture inserts were washed with PBS and incubated with DMEM containing 50 μg/ml gentamicin for 30 min at 37°C with 5% CO_2_ to remove extracellular bacteria. This is considered the 2.5-h postinfection time point. To assess intracellular replication, the monolayers were incubated in gentamicin-containing medium for an additional 3, 6, or 24 h (considered the 5.5-, 8.5-, and 26.5-h postinfection time points, respectively). The media from apical and basolateral compartments were collected for cytokine analysis. To enumerate intracellular bacteria, the monolayers were washed with DPBS and lysed using 1% Triton X-100 by gentle scraping and incubation at 37°C with 5% CO_2_ for 20 min. Serial dilutions were plated in quadruplicate on TSA plates and incubated overnight at 37°C.

### Western blot analysis.

Enteroid monolayers were washed with PBS twice and then harvested in radioimmunoprecipitation assay (RIPA) lysis buffer (Pierce) supplemented with 1× Halt protease inhibitor cocktail (Thermo Scientific) and 1× EDTA (Thermo Scientific). The lysates were freeze-thawed once in liquid nitrogen and then incubated at 4°C with end-over-end rotation for 2 h. The lysates were centrifuged at 13,000 rpm for 15 min at 4°C to pellet cellular debris. The supernatant was transferred to a fresh tube. Protein quantitation was carried out using the Bio-Rad DC protein assay kit (Bio-Rad), according to the manufacturer’s protocol.

Lysates were further solubilized in NuPAGE LDS sample buffer (Invitrogen) supplemented with 50 mM the reducing agent dithiothreitol (DTT) (Life Technologies) and heated at 95°C for 10 min. The samples were cooled to room temperature, and 20 μg of the total protein lysate was loaded into each well of a NuPAGE 3 to 8% Tris-acetate gel (Invitrogen). Electrophoresis was carried out using 1× Tris-acetate running buffer (Life Technologies). Proteins were transferred to a polyvinylidene difluoride (PVDF) membrane (Bio-Rad) using Tris-glycine transfer buffer with methanol (Bio-Rad). Membranes were blocked with 10% nonfat milk in PBS either overnight at 4°C or for 1 h at room temperature. Primary antibody incubations were carried out either overnight at 4°C or for 1 h at room temperature. Antibodies used were anti-Muc2 antibody (1:1,000) (Abcam) or anti-β-actin (1:5,000) (Invitrogen) diluted in 10% nonfat milk in PBS. The membrane was washed 3 times with PBS-Tween (0.1%) for 10 min each and then incubated with Alexa Fluor 680-conjugated secondary antibody (Thermo Fisher). Membranes were washed again and imaged using a Li-Cor Odyssey imaging system.

### Cytokine analysis.

The supernatants from apical and basolateral compartments of the enteroid monolayers were collected for cytokine analysis using a DuoSet enzyme-linked immunosorbent assay (ELISA) kit for human IL-8 (R&D Systems). The ELISA was performed according to the manufacturer’s protocol. The amount of IL-8 is reported as picograms contained in the total volume of the culture supernatant present in the apical and basolateral compartments of the enteroid monolayers at the indicated times postinfection.

### Immunofluorescence staining and confocal microscopy.

Enteroid monolayers were fixed using 4% paraformaldehyde for 30 min at room temperature and then washed three times with DPBS. Monolayers were permeabilized and blocked in a single step using PBS containing 2% bovine serum albumin (BSA), 15% fetal bovine serum (FBS), or 5% normal goat serum and 0.1% saponin for 1 h at room temperature. Monolayers were washed and removed from the transwell by carefully cutting along the edges using a scalpel. Immunostaining was performed with the following antibodies, typically for 1 h at room temperature or 4°C overnight: anti-glycoprotein 2 (MBL) and anti-ZO-1 (Invitrogen). All primary antibody dilutions were made in PBS containing 2% BSA. The monolayers were washed three times with PBS and incubated with Alexa Fluor-conjugated secondary antibodies, diluted in PBS, for 1 h at room temperature. The monolayers were washed three times and mounted in Prolong gold antifade mountant (Invitrogen) overnight at room temperature.

Imaging was carried out at the Confocal Microscopy Core at the University of Maryland, Baltimore, using a Nikon W1 spinning-disk confocal microscope. Images were captured using a 40× oil objective. Image processing was carried out using Volocity 3D image analysis software (Perkin-Elmer) or FIJI (72).

### Statistical analyses.

Statistical significance was determined using two-tailed, unpaired Student’s *t* test. For multiple comparisons, analysis of variance (ANOVA) was used with a Bonferroni posttest to determine statistical differences within specific groups. Replicates from multiple independent experiments were pooled. The number of replicates pooled from “*n*” independent experiments is reported in the figure legends. GraphPad Prism software was used for all statistical analyses.

## Supplementary Material

Supplemental file 1
