# Supplementary material for: Evaluating Shigella flexneri Pathogenesis in the Human Enteroid Model
Source: Infect Immun. 2019 Mar 25;87(4):e00740-18. doi: 10.1128/IAI.00740-18 (PMC6434113; doi:10.1128/IAI.00740-18)
Supplement: Supplemental file 1 [file IAI.00740-18-s0001.pdf]

Figure S1.

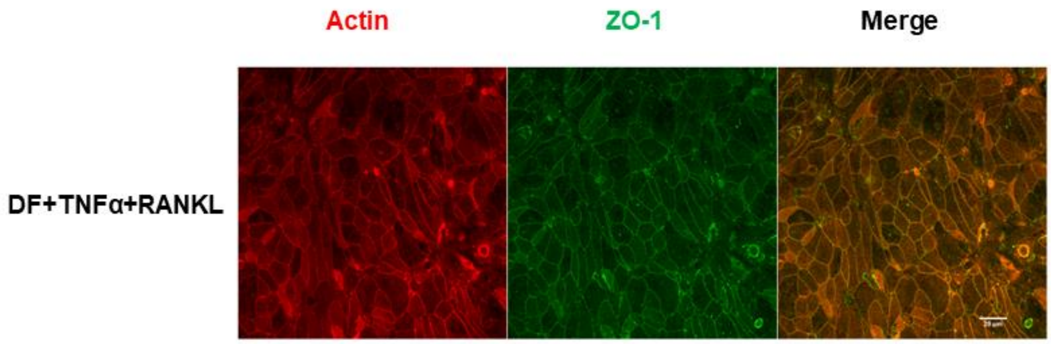

Figure S1: Tight junction protein ZO-1 immunostaining was visualized in enteroid monolayers differentiated to include M cells using confocal microscopy to confirm integrity of tight junctions. Actin in labeled with Alexa Fluor 555-conjugated Phallodin probe (red), and ZO-1 is in green.
